# Tumor Size in Early-Stage NSCLC Is a Prognostic Factor in Single Segmentectomies but Not in Multiple Segmentectomies: A Single-Center Analysis

**DOI:** 10.3390/cancers17172778

**Published:** 2025-08-26

**Authors:** Marco Chiappetta, Antonio Giulio Napolitano, Carolina Sassorossi, Dania Nachira, Filippo Lococo, Elisa Meacci, Chiara Scognamiglio, Maria Teresa Congedo, Gloria Santoro, Ettore D’Argento, Jacopo Russo, Guido Horn, Stefano Margaritora

**Affiliations:** 1 Department of Thoracic Surgery, Fondazione Policlinico Universitario “A. Gemelli” IRCCS, Università Cattolica del Sacro Cuore, 00168 Rome, Italy; marcokiaps@hotmail.it (M.C.); sassorossi.caro@gmail.com (C.S.); dania.nachira@policlinicogemelli.it (D.N.); filippo.lococo@policlinicogemelli.it (F.L.); elisa.meacci@policlinicogemelli.it (E.M.); kiasco1998@gmail.com (C.S.); mariateresa.congedo@policlinicogemelli.it (M.T.C.); stefano.margaritora@policlinicogemelli.it (S.M.); 2 Thoracic Surgery, Magna Graecia University, 88100 Catanzaro, Italy; 3 General Surgery Unit, Fondazione Policlinico Universitario Agostino Gemelli IRCCS, 00186 Rome, Italy; gloria.santoro@policlinicogemelli.it; 4 Medical Oncology, Fondazione Policlinico Universitario Agostino Gemelli IRCCS, 00186 Rome, Italy; ettore.dargento@policlinicogemelli.it (E.D.); jacopo.russo@policlinicogemelli.it (J.R.); guidohorn90@gmail.com (G.H.)

**Keywords:** segmentectomy, early stage, NSCLC, VATS

## Abstract

**Objective:** Segmentectomy has recently been accepted as a valid anatomical resection in the early stages non-small cell lung cancer, even if different segment numbers and combinations are included. The aim of this study is to analyze prognostic factors in patients who underwent segmentectomy, with particular attention to segment numbers and characteristics. **Methods:** Characteristics of patients who underwent uniportal VATS segmentectomy from 1/01/2017 to 31/12/2022 were reviewed and retrospectively analyzed. Patients with nodal involvement and/or distant metastases, tumors > 4 cm, who received neoadjuvant treatment and those who underwent completion lobectomy were excluded. Operatory and pathological reports were reviewed to collect data on surgical characteristics and pathology. Segmentectomies were categorized according to numbers of resected segments as single/multiple. Clinico-pathological characteristics, number of segments and nodal parameters were associated to overall survival (OS) using Kaplan–Meier curves. The log-rank test was used to assess differences between subgroups. A multivariable model was built using Cox-regression analysis including variables with *p*-values < 0.10 at univariable analysis. **Results:** The final analysis was conducted on 95 patients who met the inclusion criteria. Multiple segmentectomies were performed in 47 (49.4%) cases, of which 37 (39%) were complex cases. At univariable analysis, tumor size ≤ 2 cm (*p* = 0.006, HR:0.260; 95%CI 0.099–0.686) significantly correlated with OS: patients with pT ≤ 2 cm presented a 5YOS of 85.3% vs. 48.3% of patients with pT >2 cm, with multivariable-confirmed tumor size ≤ 2 cm as an independent prognostic factor (*p* = 0.004, HR:0.204; 95%CI 0.069–0.607). Considering the tumor size according to number of resected segments, patients who underwent single segmentectomy presented a significantly better survival for pT ≤ 2 cm: 5YOS 91.7% vs. 41.3% for pT > 2 cm (*p* = 0.001). Conversely, no significant differences in OS were present in multiple segmentectomy: 5YOS 78.9% vs. 77.1% (*p* = 0.700). Similarly, pT ≤ 2 cm correlated with OS in complex segmentectomy (*p* = 0.010) but not in simple segmentectomy (*p* = 0.098). **Conclusions:** Our study confirms the distinct prognosis associated with tumor dimensions in patients who underwent uniportal VATS segmentectomy. We confirmed the tumor dimension cut-off of 2 cm as a robust prognosticator in single and complex segmentectomies. However, no significant differences in survival were observed in multiple and simple segmentectomies, implying that tumors larger than 2 cm may necessitate extended resections.

## Simple Summary

Lung segmentectomy is increasingly used for early-stage non-small cell lung cancer (NSCLC), but outcomes may vary depending on tumor size and surgical complexity. This study evaluated survival in patients undergoing uniportal VATS segmentectomy, focusing on tumor size and the type of segmental resection. We found that tumors ≤ 2 cm were associated with good prognosis across all techniques, while in tumors > 2 cm, survival was preserved only when multiple or complex segmentectomies were performed. These findings may help tailor the surgical approach to tumor characteristics.

## 1. Introduction

Non-small cell lung cancer remains the leading cause of death for cancer in Western countries, with a significant impact in terms of public health management [1,2,3]. However, despite mortality not remaining negligible, different improvements in the last decades permitted new approaches to this cancer, increasing early-stage diagnoses and reducing the impact of surgical resections.

Indeed, large screening programs made it possible to intercept early-stage tumors, with the possibility of administering early treatment and subsequent survival improvement [4,5]. Similarly, the development of minimally invasive techniques made it possible to reduce the surgical impacts on patients in terms of post-operative pain, chest tubes and hospitalization duration [2,6]. Moreover, knowledge of tumor biology and its association with radiological findings permitted treatment of pre-invasive or early-stage tumors in a radical manner, also using lung-sparing strategies.

In recent years, many studies tested segmentectomy for early-stage NSCLC. However, there is still debate on its use for tumors larger than 2 cm, especially because of concerns about margins and recurrence. The JCOG0802 trial [7] showed similar overall survival for segmentectomy, but subgroup analyses suggested higher recurrence in larger tumors.

The basic and oncological indications for segmentectomy include a tumor dimension of less than 2 cm or a ground-glass/part-solid appearance and the possibility of obtaining a safe margin at least of 1–2 cm or one corresponding to the tumor diameter [8,9,10].

However, under the segmentectomy term, many possibilities are included, considering that 10 segments for each lung are present and multiple segmentectomy combinations are possible. Moreover, segmentectomy may present more technical difficulties compared to lobectomy, with the needed approach of different intersegmental planes close to different segmental bronchi and vessels.

In combining the resection of multiple segments, it is also possible to potentially obtain safe margins, saving lung parenchyma and avoiding lobectomy, but the outcomes of different segmentectomies in terms of survival are still undefined. Finally, it is not clear if increasing the resected segments number could permit a good prognosis also in tumors with dimensions greater than 2 cm. Indeed, most studies considered tumor dimensions of 2 cm or less, while indications to segmentectomy for tumors > 2 cm in size are still in doubt.

The aim of this study is to analyze the survival outcomes of patients with NSCLC who underwent segmentectomies, considering tumor characteristics and different segmentectomy classifications in p I tumors.

## 2. Methods

Data of patients who underwent segmentectomy from 1/01/2017 to 31/12/2022 at our institution were reviewed and retrospectively analyzed. Operatory and pathological reports were reviewed to collect data on surgical characteristics and pathology.

The inclusion and exclusion criteria were defined as follows:

**Inclusion criteria**

- NSCLC adenocarcinoma, squamous cell carcinoma or mixed histology;
- Age > 18 years;
- Segmentectomy;
- Complete resection;
- Dimension < 4 cm;
- Solid appearance at the CT scan;
- Uniportal VATS approach.

**Exclusion criteria:**

- Nodal involvement;
- Distant metastases;
- Neoadjuvant treatment;
- Adjuvant treatment;
- Completion lobectomy.

Pre-operative evaluation consisted in brain–thorax and abdominal CT scans with contrast, 18FDG PET scan, blood analysis and lung function testing. In patients with central tumors > 3 cm and with suspected mediastinal lymph nodes, EBUS-EUS was performed to exclude possible nodal involvement.

Segmentectomies were categorized according to the number of resected segments as single or multiple and according to the number of intersegmental planes approached as simple or complex. Simple segmentectomy was defined as only one intersegmental plane being approached, and included superior segmentectomy, upper division segmentectomy and lingular segmentectomy [11,12]. Pathological reports were reviewed and staged according to the 9th TNM edition [13].

Surgery was conducted by certified thoracic surgeons according to uniportal video-assisted thoracic surgery (VATS), as previously described by Gonzales-Rivas et al. [14]. Nodal sampling or radical mediastinal dissection according to ESTS guidelines [15] was performed, but the decision regarding the extent of lymphadenectomy was decided by the surgeon according to the tumor and patient characteristics. EBUS was performed systematically in tumors > 3 cm and with central location, while in the other cases target station biopsy was performed in case of enlarged CT scan nodes or increased 18FDG PET uptake.

Follow-up consisted of brain–thorax and abdominal CT scans, with contrast every 6 months for the first 3 years and then annually. In case of suspected recurrence, a new biopsy was attempted.

### Statistical Analysis

Overall survival was calculated from surgery to death due to any cause. Disease-free survival was calculated from surgery due to recurrence appearance or death due to any cause.

Clinico-pathological characteristics, number of segments and nodal parameters were associated to overall survival (OS) using Kaplan–Meier curves. The log-rank test was used to assess differences between subgroups.

Explanatory Descriptive Analysis (EDA) was performed to describe the dataset of studied patients. Subsequently, association analyses between the conditions of simple vs. complex segments and single vs. multiple segments were conducted for survival analysis, considering Disease-Free Survival (DFS). Conditions with a *p*-value less than or equal to 0.05 were considered indicative of a significant association. Statistical analyses were performed using the IBM SPSS Statistics for Macintosh, Version 25.00 (Armonk, NY, USA), implemented with the survival and survminer packages for the graphical representation of Kaplan–Meier curves.

A multivariable model was built using Cox-regression analysis including variables with *p*-values < 0.10 at univariable analysis.

## 3. Results

The final analysis was conducted among 95 patients that respected the inclusion and exclusion criteria. Clinical and pathological characteristics are reported in Table 1.

Multiple segmentectomies were performed in 47 (49.4%) cases, consisting of a major part of left S1–S2–S3 (23.1%) and S4–S5 (14.7%). Simple and complex segmentectomies were performed in 58 (61%) and 37 (39%) of cases, respectively. Segment classifications are reported in Table 2.

### 3.1. Characteristics of Single vs. Multiple Segments

A significant difference was observed in the distribution of the single and multiple segmentectomies based on the side of diagnosis. For patients with right-sided tumors, single vs. multiple segmentectomies resulted in 67.3% vs. 15.2% (*p* < 0.001). In contrast, for patients with left-sided tumors, 32.7% underwent single segmentectomy vs. 84.8% for multiple.

Regarding hospitalization, the single segmentectomy had a longer mean recovery time of 4.6 ± 1.8 days compared to 3.9 ± SD = 1.6 days for multiple segmentectomy (*p* = 0.033).

A significant difference was observed in the margin distance between the two conditions: mean margin distance of 5.6 ± 5.0 mm in single vs. 8.7 ± 5.6 mm in multiple segmentectomy (*p* = 0.006). Similarly, A significant difference was also observed in the total number of lymph nodes removed: 6.6 ± 5.7 vs. 4.2 ± 4.5 in single vs. multiple segmentectomies (*p* = 0.029). The same results were present when considering mediastinal removed lymph nodes: 5.1 ± 5.2, vs. 2.8 ± 3.7 in single vs. multiple segmentectomies (*p* = 0.015).

These results reflect the significant differences among resected node numbers between the right and left sides: 7.8 ± 6.2 vs. 3.9 ± 3.2 (*p* = 0.023).

### 3.2. Characteristics of Simple vs. Complex Segments

A significant association was observed between tumor side (right vs. left) and segment complexity (simple vs. complex) (*p* < 0.001). Among patients with simple segments, 78.4% were located on the right side and 21.6% on the left side. In contrast, among patients with complex segments, only 19.0% were on the right side while 81.0% were on the left side.

Patients with simple segmentectomies were more likely to have a single segmentectomy compared to those with complex segmentectomies (73.0% vs. 37.9%, *p* < 0.001).

The analysis of the pT stage distribution in the complex subgroup revealed the following findings: among patients on the right side, 9.1% were staged as T1a, 63.6% as T1b and 27.3% as T2a. No patients were classified as T1c or T2b. Meanwhile, among patients on the left side, 23.4% were T1a, 63.8% were T1b, 10.6% were T1c and 2.1% were T2b. No patients were classified as T2a.

These results show a statistically significant association (*p* = 0.015), suggesting a different distribution of pathological T stages between right and left sides, with T2a stages observed only on the right and T1c/T2b stages present only on the left.

The mean number of N2 lymph nodes removed was significantly higher in patients with simple segmentectomies compared to those with complex segmentectomies: 5.5 ± 5.3 vs. 3.1 ± 3.9 (*p* = 0.014).

The number of N2 lymph nodes removed showed a statistically significant difference between the two sides within the complex subgroup, with a higher mean number of nodes removed on the right side compared to the left side: 5.6 ± 5.8 vs. 2.5 ± 3.1 (*p* = 0.016). A comparative analysis of total and N2 lymph nodes resected was performed according to pStage, tumor side (right vs. left) and tumor size (cut-off: 2 cm), as shown in Table 3.

### 3.3. Survival Outcome

During follow-up, 17 (17.9%) patients died and 13 (14%) patients experienced a recurrence, 11 local and 2 local and distant; lung was involved in 7 cases and nodal recurrence occurred in 4 cases.

**Figure 1:**
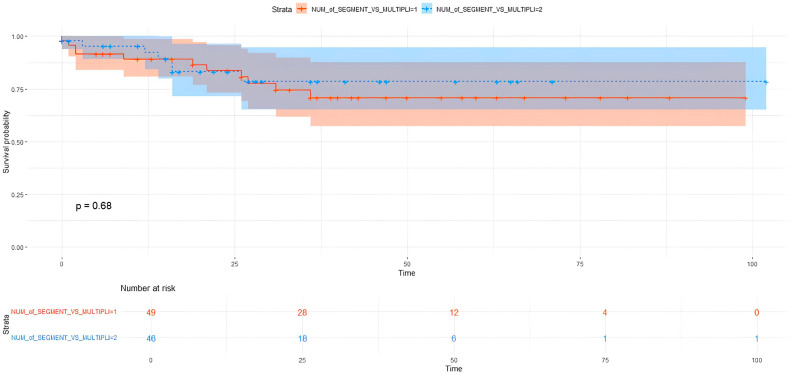
Single vs. multiple segmentectomy OS.

**Figure 2:**
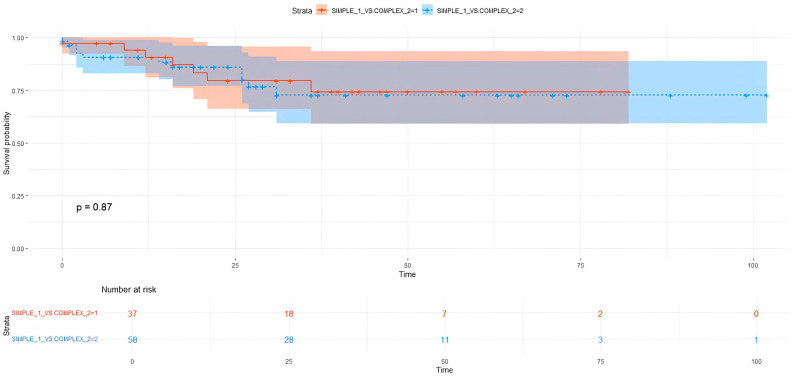
Simple vs. complex segmentectomy OS.

Comparing single vs. multiple and simple vs. complex segmentectomies, no significant differences were present in OS and DFS terms (Table 4), (Figure 1 and Figure 2).

At univariable analysis, tumor size ≤ 2 cm (*p* = 0.006, HR:0.260; 95%CI 0.099–0.686) significantly correlated with OS: patients with pT ≤ 2 cm presented a 5YOS of 85.3% vs. 48.3% of patients with pT > 2 cm (Figure 1). Multivariable analysis confirmed tumor size ≤ 2 cm as an independent prognostic factor (*p* = 0.004, HR:0.204; 95%CI 0.069–0.607).

Considering the tumor dimension according to number of resected segments, patients who underwent single segmentectomy presented significantly better survival in pT ≤ 2 cm: 5YOS 91.7% vs. 41.3% in pT > 2 cm (*p* = 0.001). Conversely, no significant differences in OS were present in multiple segmentectomy: 5YOS 78.9% vs. 77.1% (*p* = 0.700). Similarly, pT ≤ 2 cm correlated with OS in complex segmentectomy (*p* = 0.010) but not in simple segmentectomy (*p* = 0.098).

In complex segmentectomy, margin distance (*p* = 0.04) and pT dimension (*p* = 0.03) significantly correlated with OS.

Any of the analyzed variables significantly influenced the DFS in the entire cohort. In the single segmentectomy group, right-side single segmentectomy presented a significantly better DFS compared to left-side single segmentectomy (*p* = 0.036), while in the complex segmentectomy group, a significantly better DFS was present when more than 10 lymph nodes were resected (*p* = 0.024); these findings are exploratory due to the small number of DFS events.

## 4. Discussion

This study highlights that tumor size ≤ 2 cm is associated with excellent prognosis across segmentectomy types, while tumors > 2 cm show divergent outcomes depending on the extent of resection. The favorable outcome observed in multiple segmentectomies for larger tumors may be attributed to wider resection margins (>3 cm in 80% of cases). It is crucial to consider confounders such as left–right laterality bias and differences in lymph node sampling, particularly given the observed imbalance between right- and left-sided tumors in complexity and nodal yield.

With the increase in segmental resections in lung cancer management, a better definition of the indications for segmentectomy became essential to ensure the best treatment option for these patients.

As described in this study, under the “segmentectomy” definition, there are a lot of possibilities in terms of single-segment resections and combinations, varying in segment numbers and combinations.

This study confirmed that segmentectomy ensured an excellent survival outcome in tumors less than 2 cm, as previously described in other studies and trials [8,9], but we found that the kind of segment or combination may also determine the outcome.

Indeed, a significant difference in OS was present considering the 2 cm cut-off in single and complex segmentectomies, while no differences were present in multiple or simple segmentectomies.

This difference should be interpreted under different possible points of view. The first involves the possibility of larger parenchymal amounts in multiple and simple segmentectomies that may permit performing valid resections. Indeed, in our experience, margin distance results are significantly larger in multiple segmentectomies compared to single, explaining the survival difference considering the resected segment numbers according to tumor dimensions. Conversely, no significant differences in margin distance were present in simple vs. complex segmentectomies; in those groups, the significant difference according to tumor size was only present in cases of complex segmentectomies. These results may be explained by the possibility that there are more difficulties in nodal assessment when more intersegmental planes are involved, also considering that in our cohort, most recurrences resulted locally.

Our findings agree with previous literature reporting a higher recurrence rate and worse survival in cases of patients with tumors >2 cm who underwent segmentectomy [10,16,17], suggesting that this approach is valid in p IA1-2 tumors, while it is questionable in higher stages. We considered the margin distance in a different way than Lin et al. [10], who reported a significantly better survival outcome when the margin distance is larger than 2 cm.

In this study, we also found that the effectiveness of segmentectomy may vary according to different segment sides and classifications, which may influence outcomes in different ways. Indeed, as we also noticed, the number of resected lymph nodes may vary according to side and segment type, with a significantly higher number of total and mediastinal resected nodes in simple and single segmentectomies compared to multiple and complex ones. Analyzing our results, this difference was mainly related with the tumor side, with fewer resected nodes in left-sided tumors. However, this difference in lymphadenectomy did not reflect a difference in survival, perhaps due to the inclusion of patients in which radical mediastinal or lobe-specific lymphadenectomies were performed, reducing the risk of occult nodal metastases and the presence of about 80% of cases with tumors < 2 cm that presented low risk of nodal involvement. The advantage of our study, compared to others, is that all patients underwent nodal assessment, while other studies considering segmentectomy reported various rates of patients without any kind of nodal assessment or a very low number of mean resected nodes. For instance, Quintero et al. reported the mean of only four examined nodes in sublobar resections and about 20% of patients without assessment [18], and Soh et al. and Kamigaichi et al. reported 14% and 50% of segmentectomies without any kind of lymphadenectomy, respectively [16,19]. It is implicit that not performing lymphadenectomy did not yield information about nodal pathological stage, and consequently the risk to miss nodal metastases that may beneficiate adjuvant therapy and generate upstaging with consequent explanations of different prognoses among segmentectomies. Conversely, the lymphadenectomy presence in our study provides reliability to our results, focusing on the role of tumor dimensions in segmentectomies as a strong prognosticator.

On the other hand, although tumor size resulted as an independent prognostic factor, but we found that in some cases the outcome is independent of size. This concept was valid in multiple segmentectomies, perhaps due to the presence of margin distances greater than 3 cm in 80% of cases. This factor may significantly reduce the risk of recurrence on the margin, according to Dr Huang’s results [10], indicating that a margin distance > 2 cm significantly improves the survival outcome in segmentectomy. Interestingly, all these patients underwent culmen or lingual resection, confirming that in selected cases these resections should be equivalent to lobectomy.

Finally, we considered the post-operative outcomes of different segmentectomies without reporting significant differences in hospitalization and peri-operative outcomes comparing simple vs. multiple segmentectomies. These results are in contrast with the experience of Ahn et al. [11], who reported a shorter post-operative stay for complex segmentectomy. Conversely, we found that single segmentectomy had fewer hospitalization days compared to multiple segmentectomy, without differences in rates of complications among the different segmentectomy classifications.

These results confirm that segmentectomy via uniportal VATS is effective and safe, and could be adopted routinely in lung cancer management, without differences in complications among segments and with excellent results in terms of lymphadenectomy.

This study presents some limitations, due to its retrospective nature and data collection methods. Firstly, the indications to segmentectomy should be various, not only considering tumor characteristics but also the possibility of performing segmentectomy due to limited lung function. Differences in margin distance between single and multiple segmentectomies may have influenced survival outcomes, potentially favoring multiple resections. Similarly, disparities in lymph node assessment between right- and left-sided tumors could introduce bias in staging accuracy and recurrence interpretation. Another limitation pertains to indications to lymphadenectomy, which, despite being performed in all patients, were determined by the surgeon based on his experience and case evaluation. Finally, some subgroups presented limited numbers of patients, requiring confirmation of the results using larger datasets.

It should also be acknowledged that all procedures were performed using a uniportal VATS approach, which may limit the generalizability of our findings to multiport or open techniques.

On the other hand, this is one of the largest series reporting segmentectomy outcomes in uniportal VATS, also confirming the validity of this approach in complex surgeries.

## 5. Conclusions

Our study confirmed that segmentectomy provides excellent survival outcomes in early-stage NSCLC, especially for tumors ≤ 2 cm, regardless of segmentectomy type. In tumors > 2 cm, single segmentectomy was associated with worse survival, while multiple segmentectomy appeared to mitigate this disadvantage—likely due to wider margins. These findings suggest that, in selected patients, multiple segmentectomy may be a valid parenchyma-sparing option, even for tumors > 2 cm. However, no definitive conclusions can be drawn in the absence of a lobectomy control group, and the exclusive use of a uniportal VATS approach may limit the generalizability of our results to other surgical techniques. Further prospective studies are warranted to validate these observations.

## Figures and Tables

**Table 1:** Clinical and pathological characteristics.

Variable		Number (%)
**Sex**	Female Male	64 (67.3) 30 (32.7)
**Age**	Mean (SD)	69.2 (9.6)
**Smoker**	No Yes	25 (26.3) 70 (73.7)
**COPD**	No Yes	21 (22.1) 74 (77.9)
**Number of segments**	Single Multiple	49 (51.5) 46 (48.5)
**Segmentectomy**	Simple Complex	58 (61.0) 37 (39.0)
**Histology**	Adenocarcinoma Squamous cell Other	81 (85.3) 9 (9.4) 5 (5.3)
**pT**	1A 1B 1C 2A	28 (29.4) 47 (49.5) 15 (15.8) 5 (5.3)
**pT dimension**	≤2 cm >2 cm	68 (71.6) 27 (28.4)
**Margin Distance**	<1 cm 1–3 cm >3 cm	14 (14.7) 21 (22.1) 60 (63.2)
**Stations Removed**	≤2 3 or more	61 (64.2) 34 (35.8)
**Lymph nodes Removed**	<10 ≥10	77 (81.0) 18 (19.0)
**Lymph nodes Removed**	Mean (SD)	2.9 (2.4)
**N1 Removed Lymph nodes**	1 ≥2	76 (80.0) 19 (20.0)
**N2 Removed Lymph nodes**	≤3 >3	61 (64.2) 34 (35.8)
**Pleural Invasion**	No Yes	92 (96.8) 3 (3.2)

**Table 2:** Segment classification.

Segmentectomy	Included Segments	Number (%)
Simple	S6 DX/SX S1–S2–S3 SX S4–S5 SX	22 (23.1) 22 (23.1) 14 (14.7)
Complex	S1 DX/SX S2 DX/SX S3 DX S1–S2 DX/SX S1–S3 SX S6–S7SX S7 DX S7–S8–S9 DX S9–S10 SX	20 (21.0) 2 (2.1) 4 (4.4) 6 (6.6) 1 (1.0) 1 (1.0) 1 (1.0) 1 (1.0) 1 (1.0)
Single	S1 DX/SX S2 DX/SX S3 DX S6 DX/SX S7 DX	20 (21.0) 2 (2.1) 4 (4.4) 22 (23.1) 1 (1.0)
Multiple	S1–S2–S3 SX S4–S5 SX S1–S2 DX/SX S1–S3 SX S6–S7 SX S7–S8–S9 DX S9–S10 SX	22 (23.1) 14 (14.7) 6 (6.6) 1 (1.0) 1 (1.0) 1 (1.0) 1 (1.0)

**Table 3:** Comparative analysis of total vs. N2 lymph nodes resected according to pStage.

	Total Lymph Nodes Resected	N2 Lymph Nodes Resected
pStage IA1 (n = 28) IA2 (n = 48) IA3 (n = 8) IB (n = 7) IIA (n = 4)	*p*-value: 0.55	*p*-value: 0.66
Side Dx: 40 Sx: 55	*p*-value: <0.01	*p*-value: <0.01
Tumor Dimension	*p*-value: <0.01	*p*-value: <0.01

**Table 4:** Univariable analysis.

Variable		OS *p*-Value	DFS *p*-Value
**Sex**	Female Male	0.123	0.83
**Age**	Mean (SD)	**0.011**	0.12
**Smoker**	No Yes	0.775	0.83
**COPD**	No Yes	0.304	0.12
**Number of segments**	Single Multiple	0.831	0.91
**Segmentectomy**	Simple Complex	0.653	0.42
**Histology**	Adenocarcinoma Squamous cell Other	0.882	0.97
**PT**	1A 1B 1C 2A	0.085	0.89
**PT dimension**	≤2 cm >2 cm	**0.006**	0.71
**Margin Distance**	<1 cm 1–3 cm >3 cm	0.072	0.93
**Stations Removed**	≤2 3 or more	0.837	0.78
**Lymph nodes Removed**	<10 ≥10	0.211	0.86
**Lymph nodes Removed**	Mean (SD)	0.238	0.86
**N1 Removed Lymph nodes**	1 ≥2	0.126	0.16
**N2 Removed Lymph nodes**	≤3 >3	0.651	
**Pleural Invasion**	No Yes	0.384	0.99

## Data Availability

The data presented in this study are available on request from the corresponding author.
